# Sociodemographic inequalities of suicide: A population-based cohort study of adults in England and Wales 2011-2021

**DOI:** 10.23889/ijpds.v8i2.2218

**Published:** 2023-09-14

**Authors:** Isobel Ward, Katie Finning, Daniel Ayoubkhani, Katie Hendry, Louis Sharland, Vahé Nafilyan

**Affiliations:** 1 Office for National Statistics, Newport, United Kingdom; 2 University of Manchester, Manchester, United Kingdom

## Objectives

With suicide a major public health concern, it is vital research identifies predictors of suicide to support vulnerable groups who should be targeted for intervention. We use a novel linkage of 2011 Census and population level mortality data to assess which risk factors are important predictors of suicide.

## Method

Exposures of interest were identified from Census 2011 and were sex, age, ethnicity, marital status, day-to-day impairments, religion, region, National Statistics Socio-economic Classification. Our study population consisted of 35,136,917 people aged 18-to-74; there were 35,928 suicides in our study period (28/03/2011-31/12/2021), with 73.9% occurring in men. We fitted generalised linear models with a Poisson link function, with suicide being the outcome of interest. The natural logarithm of exposure time was included as an offset term. To estimate rates of suicide per 100,000 people for each level of our exposure, by sex for the average age, we calculated marginal means.

## Results

The groups with the highest rates of suicide were those who reported an impairment affecting their day-to-day activities, those who were long term unemployed or never had worked, or those who were single or separated. Comparison of minimally adjusted models with models accounting for all other characteristics identified predictors which remain important risk factors after accounting for other characteristics; day-to-day impairments were still found to increase the incidence of suicide relative to those whose activities were not impaired after adjusting for employment status. Additionally, the estimated rates of suicide remained lowest in London compared to other regions in our fully adjusted estimates. Overall, rates of suicide were higher in men compared to females across all ages, with the highest rates in 40- to 50-year-olds.

## Conclusion

The findings of this work provide novel population level insights into the risk of suicide by sociodemographic characteristics, this work should pave the way for further research exploring the interaction of factors which lead to suicide and drive policy change for targeted intervention.

